# A geminivirus-based guide RNA delivery system for CRISPR/Cas9 mediated plant genome editing

**DOI:** 10.1038/srep14926

**Published:** 2015-10-09

**Authors:** Kangquan Yin, Ting Han, Guang Liu, Tianyuan Chen, Ying Wang, Alice Yunzi L. Yu, Yule Liu

**Affiliations:** 1 Center for Plant Biology, MOE Key Laboratory of Bioinformatics, School of Life Sciences, Tsinghua University, Beijing 100084, China; 2 State Key Laboratory of Plant Genomics, Institute of Microbiology, Chinese Academy of Sciences, Beijing, China

## Abstract

CRISPR/Cas has emerged as potent genome editing technology and has successfully been applied in many organisms, including several plant species. However, delivery of genome editing reagents remains a challenge in plants. Here, we report a virus-based guide RNA (gRNA) delivery system for CRISPR/Cas9 mediated plant genome editing (VIGE) that can be used to precisely target genome locations and cause mutations. VIGE is performed by using a modified Cabbage Leaf Curl virus (CaLCuV) vector to express gRNAs in stable transgenic plants expressing Cas9. DNA sequencing confirmed VIGE of endogenous *NbPDS3* and *NbIspH* genes in non-inoculated leaves because CaLCuV can infect plants systemically. Moreover, VIGE of *NbPDS3* and *NbIspH* in newly developed leaves caused photo-bleached phenotype. These results demonstrate that geminivirus-based VIGE could be a powerful tool in plant genome editing.

Genome modification is the core and key step in genetic study of plants. It can be accomplished by random and targeted (site-specific) mutagenesis. Although there are many tools to generate random mutations, such as chemical mutagenesis by mutagens, physical mutagenesis by irradiation with fast neutrons, γ-rays or X-rays, and biological mutagenesis by transposon or T-DNA in plants, it is extremely hard to separate desired mutations from random mutations. To overcome this limitation, three approaches, including Zinc finger nucleases (ZFNs), TAL effector nucleases (TALENs) and CRISPR/Cas editing systems, have been developed to accomplish target-specific mutagenesis by inducing double stranded DNA breaks at a specific location in multiple organisms including plants, which stimulate the cellular DNA repair mechanisms including error-prone non-homologous end joining (NHEJ)^1^.

CRISPR/Cas DNA editing system was most recently developed as a new method for genome engineering. It is based on the type II CRISPR (clustered regulatory interspaced short palindromic repeats)/Cas (CRISPR associated) immune system in bacteria that protects against invading DNA viruses and/or plasmids^2^,^3^,^4^,^5^. The type II CRISPR/Cas system takes advantage of short fragments of foreign DNA-spacers that integrat into the CRIPSR loci and are subsequently processed into CRISPR RNAs (crRNAs) via transcription. The crRNAs, in turn, anneal to trans-activating crRNAs (tracrRNA) and create guide RNAs (gRNA) that direct sequence-specific cleavage and silencing of foreign invading DNA through Cas proteins. In recently developed CRISPR/Cas DNA editing system, gRNAs can direct endonuclease Cas9 to induce precise cleavage at a targeted site^2^. The CRISPR/Cas system has demonstrated the ability to achieve gene disruptions both in mammalian cells^3^,^4^ and in whole organisms, such as yeast^6^, zebrafish^7^ and worm^8^. Later, this method was quickly adopted to plants where it continued to demonstrate its applicability through transient experiment and transgenic plants^9^,^10^,^11^,^12^,^13^,^14^,^15^.

CRISPR/Cas is more advantageous than both ZFNs and TALENs because it only requires a single Cas9 nuclease that can be programmed by engineering the gRNA to direct target-specific cleavage, but it does not require elaborate design and time-consuming assembly of individual DNA-binding proteins^1^,^16^. It has been demonstrated that the CRISPR/Cas system is able to achieve efficient gene editing in plants through either transient experiment or transgenic plants^9^,^10^,^11^,^13^,^14^,^16^. In most cases, Cas9 and gRNAs are delivered into plant cells by *Agrobacterium-*mediated T-DNA transformation or physical means, such as PEG-mediated transformation of protoplast or biolistic transformation of callus. Geminiviral DNA replicon can enhance gene targeting frequencies with one to two orders of magnitude increase over conventional *Agrobacterium tumefaciens* T-DNA transformation^17^. Very recently, it was reported that *Tobacco rattle virus* (TRV), a RNA virus that replicates in cytoplasm, could deliver gRNAs into transgenic Cas9-expressing *Nicotiana benthamiana* to achieve systemic genome editing, which can be detected even in two offspring plants^18^. However, there is no DNA virus-based delivery system for Cas9-based systemic genome editing in plants.

Cabbage Leaf Curl virus (CaLCuV) is a member of the *Begomovirus* genus in the *Geminiviridae* family that replicates in nucleus^19^. It encodes seven proteins by two separate genomes: AL1 is for viral replication^20^,^21^, AL2 is a transcription activator of viral genes in late infection^22^, AL3 is a replication enhancer^23^, AL4 is a putative pathogenicity determinant and may act as post-transcriptional gene silencing (PTGS) suppressor^24^,^25^, AR1 is a coat protein, BR1 and BL1 are movement proteins^26^. The AR1 protein is required for insect transmission^27^,^28^ but not required for CaLCuV infectivity^29^. Moreover, deletion of AR1 attenuates symptoms^29^, which is an advantage for a viral vector. This modification has led to the development of CaLCuV as a virus induced gene silencing (VIGS) and miRNA expression vector^29^,^30^. CaLCuV infects a broad range of hosts within *Brassicaceae* including cabbage, cauliflower and Arabidopsis, and within *Solanaceae* including *N. benthamiana*, which extends its application in plant biotechnology.

Virus induced gene silencing (VIGS) is widely used in plant gene functional study. However, VIGS can only down-regulate gene expression, and its efficiency varies during different developmental stages. To overcome these limitations, we used a DNA virus to perform systemic gene knock-out in plants. Virus-mediated gene knockout will not have these limitations. Here, we report that *Cabbage Leaf Curl virus* (CaLCuV), a geminivirus, is able to deliver gRNA and induce systemic gene mutations in plants.

## Results

### Generation of transgenic tobacco plants expressing CAS9

CRISPR/Cas system is a two-component system and requires Cas9 nuclease and a small gRNA that targets the specific site of DNA^31^. To perform genome editing in plants, we generated stable transgenic plants that express Cas9 in *N. benthamiana*. Because the original Cas9 gene was from the prokaryote *Streptococcus pyogenes*, we synthesized a codon-optimized Cas9 (*oCas9*) based on Arabidopsis codon usage. The *oCas9* contains two nuclear localization signal (NLS) sequences at each terminus (see Supplementary Fig. S1 and Fig. S2 online). The *oCas9* should have better expression than the original *Cas9* in plants because N-terminal YFP-tagged NLS-oCas9-NLS exhibited higher expression levels than the non-codon-optimized version of Cas9 (see Supplementary Fig. S2 online). 8 independent *N. benthamiana* T1 transgenic lines were generated (see Supplementary Fig. S3 online). In all 8 transgenic lines of T1 plants, we did not observe any developmental defects in any of the developmental stages (data not shown). PCR assays indicated that *oCas9* was present in the genomes of all the transgenic lines. Western blot assays using anti-Cas9 monoclonal antibody indicated that transgenic line 8 had the highest level of oCas9 expression (see Supplementary Fig. S4 online). Thus, we chose line 8 (named KQ334 plant) for our further studies.

### Confirmation of Cas9 activity using fsGUS reporter system

**Figure 1:**
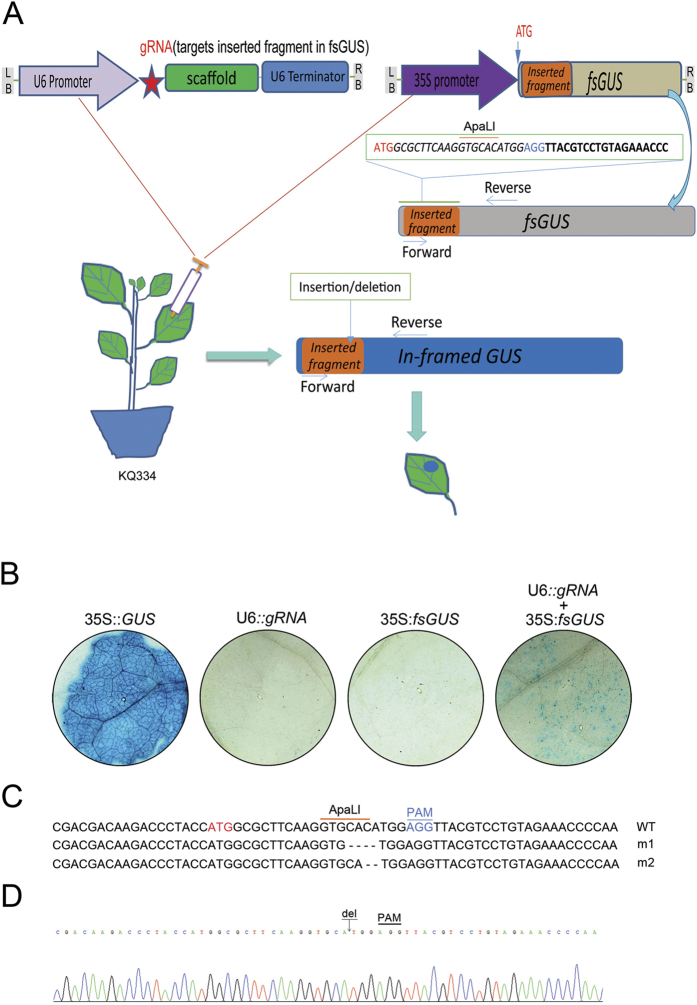
fsGUS reporter system. (**A**) Illustration of the fsGUS reporter system. A 23 bp sequence containing an ApaLI site was inserted after the start codon of the GUS gene, and the resulting sequence was designated as *fsGUS*. Due to the inserted sequence, *fsGUS* cannot be translated into a functional enzyme. *fsGUS* was driven by a CaMV35S promoter and placed in a binary vector. Another binary vector contained U6 promoter-driven gRNA targeting the first 20 bp of the inserted sequence in *fsGUS*. Two *A. tumefaciens* lines transformed with each binary vector were mixed together and infiltrated into KQ334 plant leaves. After two T-DNAs entered into the same cell, the first 20 bp of the inserted sequence in *fsGUS* was targeted by the Cas9/gRNA complex for cleavage. Repair via NHEJ will create in-dels at the cleavage site and in some cases, recover the normal coding frame for *fsGUS*, thus creating a functional GUS which can be detected by GUS staining. (**B**) GUS staining of KQ334 plant leaves. Constructs infiltrated into KQ334 plant leaves were indicated above each circle area. Blue staining only occurred in leaves infiltrated with 35S::*fsGUS* and U6p::*gRNA*. 35S::*GUS* is used as a positive control. (**C**) DNA sequences of *fsGUS* and repaired versions following cleavage by Cas9/gRNA complex. PAM was shown in blue, *ApaLI* site was shown in orange overline and start codon was shown in red. (**D**) Sanger sequencing chromatograph of the deletion from m2 in C, which results in an in-frame *GUS* gene. “del” represents deletion.

We used a frame-shift GUS (fsGUS) reporter system to test whether the transgenic Cas9 line 8 retains nuclease activity. In this system, a 23 bp sequence with a PAM (AGG) at the end is inserted downstream of the start codon of the *GUS* gene, which causes a frame shift of the *GUS* gene^15^. Once gRNA targets the first 20 bp of the inserted sequence, Cas9 will be recruited to the target site and cleave target DNA 3 bp upstream of the PAM motif. Cleavage of the target DNA will be mostly repaired by NHEJ. This DNA-repair mechanism often causes small insertions, deletions, and/or substitutions at the cleavage site. In some cases, these alterations will correct the abnormal frame of *fsGUS* gene, producing a functional GUS, which can be detected by colored deposition in plant tissue (Fig. 1). For easy detection of editing events, an *ApaLI* site was incorporated into the 23 bp inserted sequence near to PAM (Fig. 1A).

To detect whether KQ334 plants retained Cas9’s activity, the leaves were co-infiltrated with a mixture of two *A. tumefaciens* strains: one harboring the *fsGUS* gene driven by a 35S promoter and the other one containing an *Arabidopsis U6-26* gene promoter (U6 promoter)-driven *gRNA* gene containing a 20 bp sequence that matches the first 20 bp of the sequence inserted into the *fsGUS* gene (Fig. 1A). At 56 hours post infiltration, leaves were harvested and vacuum-infiltrated by staining solution containing 5-bromo-4-chloro-3-indolyl β-D-glucuronide (X-Gluc). As expected, no GUS expression was observed in all leaves agroinfiltrated with the *fsGUS* gene alone. However, GUS expression was observed in leaves co-infiltrated with *fsGUS* and *gRNA* genes. The leaves co-infiltrated with *fsGUS* and *gRNA* showed weaker coloration compared to that infiltrated with 35S::*GUS* construct (Fig. 1B). We extracted genomic DNA from leaves with co-infiltration, followed by ApaLI digestion and subsequent PCR amplification. PCR products were then cloned and sequenced. We detected a 4 bp and a 2 bp deletion before the PAM motif (Fig. 1C,D). Taken together, these experiments demonstrate that KQ334 plants retain Cas9 activity and cleave DNA under the guidance of gRNA.

### VIGE of *NbPDS* gene

We tested if CaLCuV could be used for geminivirus-based gRNA delivery system for CRISPR/Cas9 mediated plant genome editing (VIGE) in KQ334 plants by using two CaLCuV-based T-DNA vectors pCVA and pCVB^30^. To investigate whether VIGE can target endogenous genes and guide Cas9 cleavage at the target site, we chose a 20 bp sequence^10^ that target *NbPDS*’s first exon. Phytoene desaturase (designated PDS) is a key enzyme in the carotenoids biosynthetic pathway to convert phytoene to carotenoids in plants^32^. *PDS* gene often exists in plant genomes as a single copy gene^33^. Loss of function for *PDS* gene causes photobleaching and has been used widely as a marker gene in VIGS in many plant species^34^,^35^.

**Figure 2:**
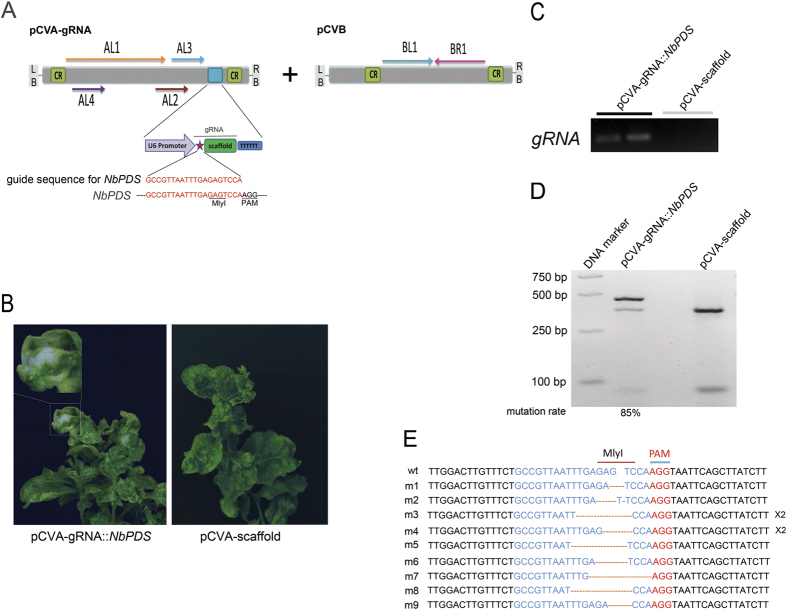
VIGE in *N. benthamiana*. (**A**) Schematic diagram of T-DNA region in VIGE vectors pCVA-gRNA and pCVB. Arabidopsis *U6-26* gene promoter (U6 promoter) in pCVA-gRNA is used to direct the expression of gRNA for VIGE in transgenic Cas9-expressing plants. *NbPDS*-targeting guide sequence was shown in red. CR, common region. (**B**) Agroinfection of transgenic Cas9-expressing *N. benthamiana* (KQ334) plants with pCVB and pCVA-gRNA::*NbPDS* (left), but not pCVB and pCVA-scaffold (right), caused photobleached phenotype. Pictures were taken at 12 weeks post agroinfiltration (wpi). (**C**) RT-PCR to show that gRNA is expressed in pCVA-gRNA:*NbPDS/*pCVB co-infiltration KQ334 plants but not in pCVA-scaffold/pCVB co-infiltration KQ334 plants. (**D**) VIGE showed high editing efficiency in pCVA-gRNA::*NbPDS*/pCVB co-infiltrated KQ334 plants. The *NbPDS* locus was PCR amplified using genomic DNA from photobleached leaf area of pCVA-gRNA::*NbPDS*/pCVB co-infiltrated KQ334 plant as well as from control plant. PCR product was then digested with MlyI and subsequently run on a 2% gel. The mutation rate was calculated by dividing the intensity of the uncut band by the intensity of all bands in the lane. (**E**) DNA sequence of wild type (wt) and mutant versions of *NbPDS* caused by cleavage by Cas9/gRNA complex and DNA repair. PAM was shown in red, *MlyI* site was shown in dark red overline and gRNA target site was shown in blue. x2 means two same clones.

To express gRNA by viral vector, we inserted a cassette containing a U6 promoter and gRNA::*NbPDS* (Fig. 2A and see Supplementary Fig. S1 online) into pCVA to generate pCVA-gRNA::*NbPDS*. Another cassette containing only the U6 promoter and scaffold was cloned into pCVA to generate pCVA-scaffold as a negative control. We inoculated KQ334 plants with a mixture of two *A. tumefaciens* strains containing pCVB and pCVA-gRNA::*NbPDS* or pCVA-scaffold. Because CaLCuV is a DNA virus that replicates in the nucleus^30^, gRNA::*NbPDS* is expected to be produced in nucleus under the control of the U6 promoter in the CaLCuV vector. Furthermore, since *NbPDS* encodes an enzyme in carotenoid biosynthesis and its silencing causes a photobleached phenotype in plants^30^, we expect that genome editing in *NbPDS* will cause a bleached phenotype in non-inoculated leaves of KQ334 plants due to quick and systemic virus infection. Indeed, we observed that 30% of pCVA-gRNA::*NbPDS*/pCVB co-infiltrated KQ334 plants showed photobleaching in leaves by three weeks post infiltration (wpi) (see Supplementary Fig. S5 online), during which the photobleached phenotype became most visible at 12 wpi (Fig. 2B). However, KQ334 plants infiltrated with pCVA-scaffold/pCVB did not show photobleached phenotype (Fig. 2B). Furthermore, wildtype *N. benthamiana* plants without the Cas9 transgene (wt plants) co-infiltrated with pCVA-gRNA::*NbPDS*/pCVB showed leaf curl viral symptom but no photobleached phenotype (see Supplementary Fig. S6 online). These results suggest that the photobleached phenotype on pCVA-gRNA::*NbPDS*/pCVB co-infiltrated KQ334 plants was not caused by CaLCuV-mediated VIGS via a 20 bp guide sequence sharing identity with *NbPDS*, which is consistent with previous research that discovered siRNA-mediated VIGS using CaLCuV with *PDS* insertions less than 40 bp did not result in any VIGS phenotype^30^. We further confirmed by RT-PCR that *NbPDS*-targeting gRNAs were expressed in systemic leaves of pCVA-gRNA::*NbPDS*/pCVB co-infected KQ334 plants but not pCVA-scaffold/pCVB control KQ334 plants (Fig. 2C).

To investigate whether endogenous *NbPDS* gene was edited, we extract pCVA-gRNA::*NbPDS*/pCVB co-infected KQ334 plants as well as the control KQ334 plants genomic DNA from systemic leaf tissues with visible bleached phenotype. Next, we performed PCR amplification of *PDS* DNA fragments and a subsequent digestion with MlyI to eliminate the possible influence of unedited *NbPDS* gene, since one MlyI cut site exists in the gRNA target sequence of *NbPDS* and will be eliminated by gene editing. As expected, the genomic DNA from both the control and pCVA-gRNA::*NbPDS*/pCVB co-infiltrated KQ334 plants gave the expected 450 bp PCR bands. PCR products from the control KQ334 plants (as a negative control) were easily completely digested with MlyI and gave two DNA bands. However, most PCR products from plants co-agroinfiltrated with pCVA-gRNA::*NbPDS*/pCVB could not be digested with MlyI due to gene editing, and the mutation rate is estimated as 85% (Fig. 2D). Furthermore, the cloned PCR product could not be digested with MlyI—following DNA sequencing of all 11 clones, we found that each clone had indels in *NbPDS* (Fig. 2E). All the indels can be grouped into nine different mutation types ranging from 1–8 bp deletions and 1 bp insertions (Fig. 2E), suggesting that genome editing in *NbPDS* occurs in KQ334 plants co-agroinfiltrated with pCVA-gRNA::*NbPDS*/pCVB. These results clearly suggest that our CaLCuV vector can be used to perform virus-based genome editing in plants.

### VIGE of *NbIspH* gene

**Figure 3:**
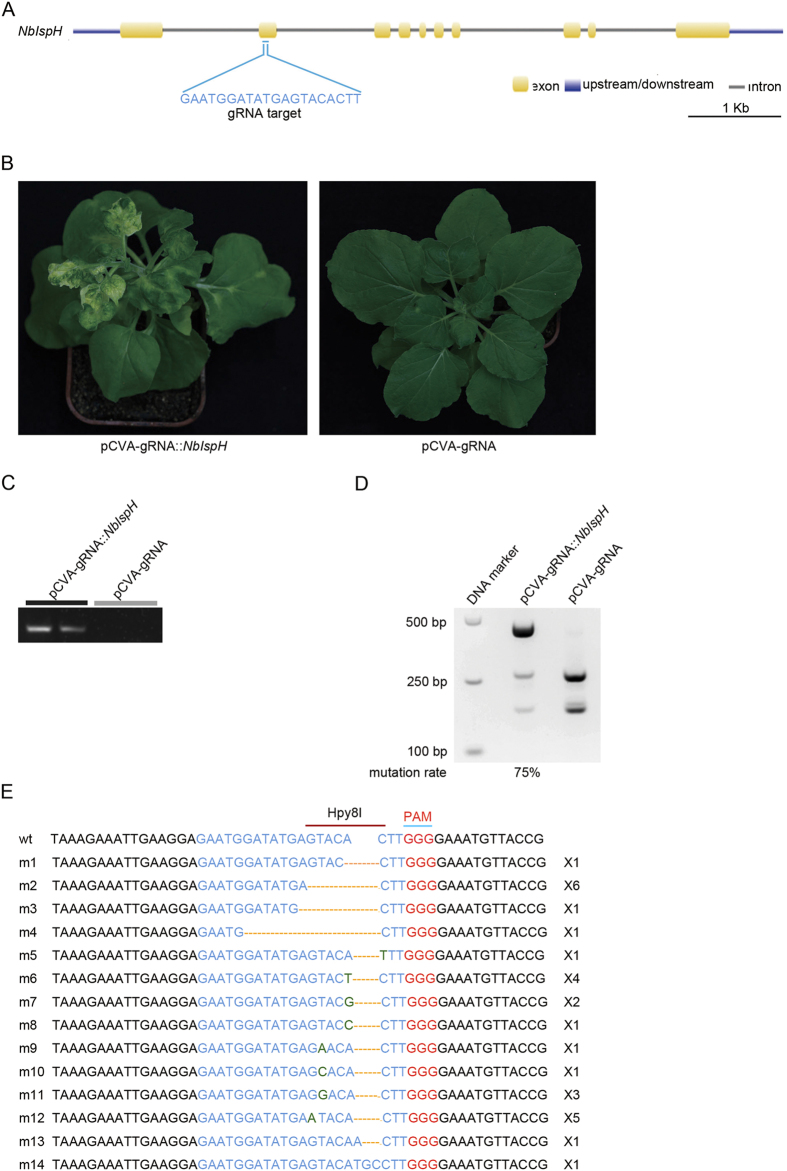
VIGE of *NbIspH* in *N. benthamiana*. (**A**) Gene structure of *NbIspH* gene and positon of gRNA target in *NbIspH* gene. Exon, upstream or downstream region and intron of *NbIspH* gene were shown in different colors and displayed in scale. gRNA target is in the second exon of *NbIspH* gene, and the sequence was shown in detail under the corresponding exon. (**B**) Agroinfection of transgenic Cas9-expressing *N. benthamiana* (KQ334) plants with pCVB and pCVA-gRNA::*NbIspH* (left), but not pCVB and pCVA-scaffold (right), caused photobleached phenotype. Pictures were taken at 3 wpi. (**C**) gRNA expresses in pCVA-gRNA:*NbIspH/*pCVB co-infiltration KQ334 plants but not in pCVA-scaffold/pCVB co-infiltration KQ334 plants. (**D**) VIGE of *NbIspH* showed high editing efficiency in pCVA-gRNA::*NbIspH*/pCVB co-infiltrated KQ334 plants. The *NbIspH* locus was PCR amplified using genomic DNA from photobleached leaf areas of pCVA-gRNA::*NbIspH*/pCVB co-infiltrated KQ334 plants as well as control plants. PCR product was then digested with Hpy8I and subsequently run on a 2% gel. The mutation rate was calculated by dividing the intensity of uncut band by the intensity of all bands in the lane. (**E**) DNA sequence of wild type (wt) and mutant versions of *NbIspH* caused by VIGE of *NbIspH* gene. gRNA target sequences were shown in blue. PAM was shown in red, *Hpy8I* site was shown in dark red overline and gRNA target site was shown in blue. Substitution nucleotides were shown in green. x2 means two same clones.

To further confirm the functionality of VIGE in knocking out endogenous genes, we chose a 20 bp sequence that targets *NbIspH*’s second exon (Fig. 3A). *NbIspH* encodes an isopentenyl/dimethylallyl diphosphate synthase (IDDS) involved in the plastid nonmevalonate pathway of isoprenoid biosynthesis that converts (E)-4-hydroxy-3-methylbut-2-enyl diphosphate (HMBPP) to isopentenyl diphosphate (IPP) and dimethylallyl diphosphate (DMAPP)^36^,^37^. Both null mutant of *NbIspH* in Arabidopsis and VIGS of *NbIspH* in *N. benthamiana* showed albino phenotype^37^,^38^.

To perform VIGE of the *NbIspH* gene, pCVA-gRNA was first created by inserting a cassette containing a U6 promoter and gRNA::*NbIspH* into pCVA. The construct pCVA-scaffold was taken as a negative control. We inoculated KQ334 plants with a mixture of two *A. tumefaciens* strains containing pCVB and pCVA-gRNA::*NbIspH* or pCVA-scaffold. We observed that 50% of pCVA-gRNA::*NbIspH*/pCVB co-infiltrated KQ334 plants showed photobleaching in leaves at three wpi (Fig. 3B). However, KQ334 plants infiltrated with pCVA-scaffold/pCVB did not show photobleached phenotype (Fig. 3B). Furthermore, wildtype *N. benthamiana* plants without the Cas9 transgene (wt plants) co-infiltrated with pCVA-gRNA::*NbIspH*/pCVB showed normal viral symptoms without photobleached phenotype (see Supplementary Fig. S7 online). We further confirmed that gRNAs were expressed in systemic leaves of pCVA-gRNA::*NbIspH*/pCVB co-infected KQ334 plants but not pCVA-scaffold/pCVB control KQ334 plants through RT-PCR (Fig. 3C).

To investigate whether endogenous *NbIspH* gene was edited, we adopted a similar strategy previously used for *NbPDS* and took advantage of the *Hpy8I* site residing in a region of the *NbIspH* gene targeted by gRNA::*NbIspH.* We extracted genomic DNA from systemic leaf tissues with bleached phenotype, from both pCVA-gRNA::*NbIspH* /pCVB co-infected KQ334 plants as well as from control KQ334 plants, then performed PCR amplification of the *NbIspH* DNA fragment. Finally, a digestion using Hpy8I was performed to eliminate the possible influence of unedited *NbPDS* gene. The genomic DNA from both control and pCVA-gRNA::*NbIspH* /pCVB co-infiltrated KQ334 plants gave the expected 487 bp PCR bands. PCR products from control KQ334 plants (as a negative control) were completely digested with Hpy8I and gave two DNA bands. However, most PCR products from plants co-agroinfiltrated with pCVA-gRNA::*NbIspH*/pCVB could not be digested with Hpy8I due to gene editing, and the mutation rate was estimated as 75% (Fig. 3D). Furthermore, we cloned PCR products that could not be digested with Hpy8I. 29 clones were sent for DNA sequencing to investigate the editing events in *NbIspH* (Fig. 3E). We found that all clones had indels in *NbIspH.* All the indels can be grouped into 14 different mutation types ranging from 1–6 bp deletions, 1–3 bp insertions and 1 bp substitutions (Fig. 3E), suggesting that genome editing in *NbIspH* occurs in KQ334 plants co-agroinfiltrated with pCVA-gRNA::*NbIspH*/pCVB.

## Discussion

In this report, we have constructed a stable transgenic plant that overexpresses Cas9 - KQ334. Using an fsGUS reporter system, we confirmed the activity of Cas9 in transgenic plants. Using this stable transgenic plant, we showed that the modified CaLCuV vector can be used to express gRNAs and edit target genes in plant genomes. To the best of our knowledge, this is the first report to use geminivirus to induce targeted edition of endogenous genes in systemic leaves of plants.

Use of geminiviruses for gene targeting has long been recognized, but the genome size restraint has hindered the development of tools using full viruses for genome engineering. For some bipartite begomoviruses, the deletion of coat protein does not interfere with their normal infection, and has been used to insert foreign sequence up to 800 nt^39^. Although this capacity is not suitable for expression of nuclease such as ZFN, TALEN or Cas9, it is enough to express gRNA. Consequently, we generated a stable transgenic plant that expresses a plant codon-optimized version of Cas9 and replaces CaLCuV’s CP with gRNA. This exchange is driven by a U6 promoter to express gRNA. This strategy eliminates the cargo constraints of CaLCuV and makes it possible to perform targeted genome engineering. Initially, like other reports^9^,^15^, we used the complete version of the U6 promoter and U6 terminator to drive and terminate gRNA expression in the T-DNA vector of our fsGUS reporter system. However, the complete version of the U6 promoter and terminator in addition to the gRNA had a sum length of nearly 1000 bp, which exceeded the limit of CaLCuV’s cargo capacity but had potential of recombination to smaller variable sizes. We deleted the U6 terminator to reduce the insertion size, and found that the U6 promoter still worked well for expressing gRNA, suggesting that U6 terminator’s function can be substituted by poly Ts. The poly Ts at the end of the gRNA scaffold could potentially serve as this terminator. This observation was consistent with ones found in mammalian cells^4^. Easy construction and multiplexed genome engineering are two great advantages of CRISPR/Cas technology when compared to ZFN and TALEN; however, genome size constraints of geminiviruses hindered the application of multiplexed genome engineering. To overcome this problem, future modifications may include: (1) finding a shorter version of the U6 promoter that significantly reduces the length of the promoter while retaining its activity; (2) using ribozyme or tRNA to help express multiple gRNAs under the same modified U6 promoter^40^,^41^; (3) using other promoters like H1 promoter^42^.

Very recently, TRV was reported to be able to deliver gRNAs into transgenic Cas9-expressing *N. benthamiana* to achieve systemic genome editing, which can be detected even in 2 offspring plants^18^. Differing from TRV’s characteristic of replicating in the cytoplasm, CaLCV replicates and can express gRNA in the nucleus which coincidentally is where genome editing occurs. This may explain why our VIGE showed very high efficiency of genome editing in this study.

It is claimed that TRV-based VIGE can be transmitted to next generation^18^. One possible weak point of our system is the lack of inheritance of mutations. In the reported TRV system, the mutation rate is less than 15% in seedlings of two offspring plants^18^. However, the mutation rate should be at least 50% for individual allo-tetraploid *N. benthamiana* plants with two different indels or 25% for individual plants with one indel. Thus, there is no sound evidence to show that the TRV system can really transmit mutations to next generation. It will be a challenge to develop virus-based genome editing systems that can really transmit mutations to next generation.

Compared to traditional VIGS, our CaLCuV-based VIGE has two advantages. First, conventional VIGS uses a fragment of the targeted gene to produce siRNAs which silence the corresponding target gene, but also cause non-specific silencing especially for highly homologous genes^43^,^44^,^45^. In contrast, CRISPR/Cas based VIGE can target a specific gene and cause gene knock out after DSB is repaired by NHEJ, thus CRISPR/Cas-basesd VIGE can be used to study an individual gene’s function. Although the CRISPR/Cas system has been reported to have high off-target activity in mammalian cells, it has undetectable off-target consequences in Arabidopsis, a model plant^46^, raising its applicability in plant genetics. Second, VIGS requires cloning a fragment of the targeted gene by PCR, while VIGE only needs to synthesize a 20 bp target sequence into gRNA, providing an effective way of establishing a high-throughput platform for genome wide gene function analysis. However, the current version of VIGE also has drawbacks compared to VIGS. siRNAs produced by VIGS (e.g. TRV based VIGS) can spread to all plant tissue, even meristems. VIGE was restricted by the movement of its carrier, CaLCuV, and therefore could not move to all tissues, including the meristem of plants. In addition, Geminiviruses often exist in mature plant cells which do not divide. Taken together, these may explain why only a small portion of plants co-infiltrated with pCVA-gRNA::*NbPDS3*/pCVB showed restricted areas of photobleaching. However, we found that our VIGE can achieve very high mutation rates. Moreover, a geminivirus is excluded from regeneration during a plant’s regeneration processes—offspring plants will not carry any geminivirus^17^. Thus, it is possible to regenerate mutated plants from systemic tissues without the need of antibiotic selection and further gene transformation, and the mutated regenerated plants will not have extra T-DNA insertions besides Cas9. This may be useful for crops that are difficult to transform.

Geminiviruses have a wide range of hosts including monocots and dicots. Although our method was based on CaLCuV, which belongs to the genus *Begomovirus*, it can be simply applied to other geminiviruses vectors, such as *Tomato golden mosaic virus* (TGMV)^47^, *African cassava mosaic virus* (ACMV)^48^, *Maize streak virus* (MSV)^49^ and *Bean yellow dwarf virus* (BeYDV)^50^. As we know, many non-model plants and some model crop plants (such as maize, cotton) are difficult to transform, which hinders their reverse genetic study. With the advancement of sequencing and reduction in cost, more and more genomes of non-model plants will be uncovered quickly. Together, VIGE approach is a promising method to enable efficient genome engineering for many plants.

## Materials and Methods

### Codon optimization of Cas9

Cas9 used in this study was originally from *S. pyogenes*. To overcome its low expression level caused by codon usage differences between bacteria and plants, we optimized the *Cas9* gene according to *Arabidopsis’*s codon preference. To ensure nuclear localization of Cas9, we attached two nuclear localization signals (NLS) at its N terminal and C terminal ends respectively. We also optimized codons for two NLSs. Codon-optimized *Cas9* with two *NLS*s was synthesized and cloned into pUC19 by GeneScript to generate PUC19-*NLS-oCas9-NLS*. The codon-optimized *Cas9* with two *NLS*s is named *oCas9,* with “o’ representing “codon optimized”.

### Plasmid construction

To generate a construct for constitutive expression of *oCas9*, *oCas9* was PCR amplified using pUC19-*NLS-oCas9-NLS* as a template with primer pairs oYK465 and oYK468 (see Supplementary Table S1 online) and subsequently cloned into pJG081, a T-DNA vector containing CaMV 35S promoters with duplicated enhancers, to generate pKQ334 by ligation independent cloning (LIC) technique^51^.

To express a non-functional *GUS* gene in plants, we removed its start codon and added a short sequence of an ApaLI recognition site described in Jiang *et al.*^15^ before the start codon to stop *GUS* gene translation in advance. We designated the modified *GUS* gene as *fsGUS*. *fsGUS* was PCR amplified using primers oYK618 and oYK619 and cloned into pJG081 by LIC technique.

To drive gRNA expression in plants, we used a U6 promoter and terminator from *Arabidopsis*. U6 promoter was PCR amplified with primer pairs oYK550 and oYK551, and U6 termination was PCR amplified with primer pairs oYK552 and oYK553. gRNA scaffold was PCR amplified using a synthetic oligo encoding gRNA scaffold as the template with primer pairs oYK554 and oYK555. The resulting gRNA scaffold has a 20 bp overlap with both the U6 promoter and U6 terminator. U6 promoter, gRNA scaffold and U6 terminator were assembled by fusion PCR using primers oYK550 and oYK553. Fused PCR products were A-tailed by *Taq* DNA polymerase and then cloned into pMD18-T (Takara) to generate pT-U6p-scaffold-U6t, which was used as the template for generating gRNAs targeting different genomic regions. To generate pT-U6p-gRNA-scaffold-U6t containing various gRNAs, PCR mutagenesis was performed using pT-U6p-scaffold-U6t as template with two primers: one containing a 20 nt guide sequence and extending a 23 bp sequence that matches the first 23 bp of the scaffold and the other one containing a 20 nt sequence complementary to the guide sequence and a 23 bp antisense sequence of 3′-terminus of the U6 promoter. PCR products were digested with DpnI and directly transformed into DMT cells (Transgene, BJ) to generate pT-U6p-gRNA-scaffold-U6t by DNA repair in E. coli. U6p- gRNA:fsGUS-U6t was generated by using primer pair oYK615 and oYK616, and U6p- gRNA::*NbPDS*-U6t was generated by using primer pair oYK1056 and oYK1057. U6p- gRNA::*NbIspH*-U6t was generated by using primer pair oYK1380 and oYK1381. U6p-gRNA-scaffold-U6t cassette was cut with SacI and PstI, cloned into pCAMBIA2300 (GenBank accession number AF234315) to generate p2300-gRNA.

pCVA and pCVB were previously described^30^. To generate pCVA-scaffold, gRNA scaffold was PCR amplified from pT-U6p-scaffold-U6t using primers oYK1013 and oYK1192, and then cloned into *KpnI-XbaI* sites of pCVA. To generate pCVA-gRNA::*NbPDS* and pCVA-gRNA::*NbIspH*, gRNA::*NbPDS* and gRNA::*NbIspH* were PCR amplified using primers oYK1191 and oYK1192 from pT-U6p-gRNA::*NbPDS*-scaffold-U6t and pT-U6p-gRNA::*NbIspH*-scaffold-U6t respectively, and then cloned into *KpnI-XbaI* sites of pCVA.

### Generation of transgenic oCas9-expressing plant

Leaves of 4–8 weeks old *N.benthamina* plants were harvested and surface sterilized by immersion into 20% (v/v) NaClO with 0.1% (v/v) Tween 20 for 10 minutes. Leaves were then rinsed in sterilized water with 0.1% (v/v) Tween 20 for 4 times. Surface sterilized leaves were cut into 1 cm squares which were then incubate with pre-cultured agrobacteria containing pKQ334 diluted in liquid transformation medium (1x Murashige and Skoog basal salt mixture, 3% Sucrose, 2.0 mg/L 6-BA, 0.2 mg/l NAA, pH5.8) with an OD at 0.6 for 10 minutes at room temperature. Leaf squares were then put onto solid transformation medium (1x Murashige and Skoog basal salt mixture, 3% Sucrose, 2.0 mg/L 6-BA, 0.2 mg/l NAA, 1% (w/v) agar, pH5.8) in dark. After 2 days, leaf discs were moved to selective induction medium (1x Murashige and Skoog basal salt mixture, 3% Sucrose, 2.0 mg/L 6-BA, 0.2 mg/l NAA, 1% (w/v) agar, 25 mg/L Hygromicin, 200 mg/L Timentin, pH5.8). Explants were moved to fresh selective induction medium every 2 weeks until shoots emerged. Shoots were cut from explants and moved to root induction medium (1x Murashige and Skoog basal salt mixture, 3% Sucrose, 1% (w/v) agar, 25 mg/L Hygromicin, 200 mg/L Timentin, pH5.8). Transgenic plants with roots were transferred into soil and covered with plastic membrane to keep moisture. After 2–3 weeks, plastic membranes were removed for plant normal growth. 4 weeks later, seeds can be collected from transgenic plants.

### Plant growth, transient expression and virus inoculation

*N. benthamiana* plants were grown in growth chambers at 25 °C under a 16-h-light/8-h-dark cycle with 60% humidity. For transient expression and virus inoculation, pCVA, pCVB, p2300-gRNA and their derivatives were first introduced into *A. tumefaciens* strain GV3101. The *Agrobacterium* cultures were inoculated in a 5 mL Luria-Bertani medium containing appropriate antibiotics (50 mg L^−1^ rifampicin, and 50 mg L^−1^ kanamycin) and grown overnight in a 28 °C shaker. *Agrobacterium* cultures were harvested and resuspended in infiltration buffer (10 mM MgCl_2_, 10 mM MES, and 200 μM acetosyringone), adjusted to an optical density at 600 nm of 1.0 and incubated at room temperature for 3 to 4 h before infiltration. For transient expression, *Agrobacterium* cultures containing p2300-gRNA derivatives were infiltrated into leaves of six-leaf stage plants using a 1-mL syringe. For virus inoculation, *Agrobacterium* cultures containing pCVB and pCVA or their derivatives were mixed at a 1:1 ratio and infiltrated into petioles of six to eight-leaf stage plants using a 1-mL syringe.

### Western blotting and immunodetection

To extract total plant proteins, 200 μl 2x lysis buffer with dye (4% SDS, 20% glycerol, 120 mM Tris-HCl pH 6.8, 10% β-mercaptoethanol, 0.02% bromophenol blue.) was added to 0.4 g leaf material. Boiled samples were centrifuged to remove cell debris and then run on a 10% SDS-PAGE gel and transferred to PVDF membrane. The Cas9 protein was detected with the anti-Cas9 monoclonal antibody (Diagenode, USA).

### Semi-quantitative RT-PCR

Expression of gRNA was analyzed by semi-quantitative RT-PCR. Reverse transcription was performed with the M-MLV Reverse Transcriptase (TIANGEN Ltd, Beijing) using equal total RNA extracted from systemic leaves of pCVA-gRNA::*NbPDS*/pCVB infected KQ334 plants and pCVA-scaffold/pCVB infected KQ334 plants with primer oYK1203 . Primer oYK1202 and oYK1203 were used to amplify gRNA:*NbPDS*. Primer oYK1483 and oYK1203 were used to amplify gRNA:*NbIspH*. The thermal cycling conditions were optimized to 95 °C for 2 min, followed by 30 cycles at 94 °C for 30 s, 58 °C for 30 s and 72 °C for 30 s and a final extension of 5 min at 72 °C. The PCR product was resolved on a 1.5% agarose gel and visualized by Ex-Red dye staining.

### Detection of Cas9/gRNA mediated mutations in plant genomic DNA

For fsGUS editing detection, genomic DNA was extracted from infiltrated leaves of KQ334 plants using Plant DNAsecure Plant Kit (TIANGEN Ltd, Beijing), and resuspended in 100 μL TE buffer (10 mM Tris/1 mM EDTA, pH 8.0). 300 ng genomic DNA was digested with ApaLI in a 50 μl volume, which were then purified by Multifunctional DNA Purification Kit (BioMED, Beijing). GUS DNA fragments were PCR amplified using purified DNA as template with primers oYK700 and oYK701, and cloned into pMD18-T vector (Takara), followed by DNA sequencing to check mutations. For detection of *NbPDS* editing, genomic DNA extracted from systemic leaves was used as PCR template to amplify *NbPDS* locus with primers PDS_MlyIF and PDS_MlyIR^10^ and *EasyTaq* DNA polymerase (Transgene, Beijing). PCR product was purified and digested with MlyI and run on a 2% agarose gel. Mutation rate was estimated by ImageJ (NCBI). The uncut DNA band was gel purified by DNA purification kit (BioMed, Beijing), subsequently cloned into pMD18-T vector (Takara). DNA sequencing was used to check mutations.

### GUS staining

GUS staining procedures followed protocol described in Kabbage *et al.*^52^.

## Additional Information

**How to cite this article**: Yin, K. *et al.* A geminivirus-based guide RNA delivery system for CRISPR/Cas9 mediated plant genome editing. *Sci. Rep.* **5**, 14926; doi: 10.1038/srep14926 (2015).

## Supplementary Material

Supplementary Information
