# Supplementary material for: A geminivirus-based guide RNA delivery system for CRISPR/Cas9 mediated plant genome editing
Source: Sci Rep. 2015 Oct 9;5:14926. doi: 10.1038/srep14926 (PMC4598821; doi:10.1038/srep14926)
Supplement: Supplementary Information [file srep14926-s1.pdf]

A geminivirus-based guide RNA delivery system for CRISPR/Cas9 mediated plant genome editing

Kangquan Yin[1, 2], Ting Han[1], Guang Liu[1], Tianyuan Chen[1], Ying Wang[1], Alice Yunzi L. Yu[1, 3] and Yule Liu[1]*

[1] Center for Plant Biology, MOE Key Laboratory of Bioinformatics, School of Life Sciences, Tsinghua University, Beijing 100084, China

[2] State Key Laboratory of Plant Genomics, Institute of Microbiology, Chinese Academy of Sciences, Beijing, China.

[3] Alice Yunzi L. Yu is a summer intern from University of North Carolina at Chapel Hill

*Corresponding author:

Name Yule Liu

Telephone +86-10-62794013

Fax +86-10-62794013

E-mail yuleliu@mail.tsinghua.edu.cn

Supplementary information

Table of contents:

Supplementary figures

Supplementary table

A.

>NLS-oCas9-NLS

atggattacaaggatcacgatggagattacaaagatcacgatatagattacaaagatgatgatgataaaatggcacctaagaagaaaagaaaggttggaattcatggtgtgcctgctgcagataaga

agtacagtataggacttgatatcggtaccaactctgttggatgggctgtgatcactgatgaatataaggttcctagtaagaaattcaaggtgctcggtaacacagatagacactctattaagaagaatctt

ataggagctcttttgtttgattcaggtgaaacagctgaggcaaccagacttaaaaggacagcaagaaggagatacaccaggagaaagaacagaatttgttatttgcaagaaatcttctctaatgagatg

gctaaggttgatgattcatttttccatagattagaagagagttttcttgtggaagaggataagaaacatgaaagacaccctatattcggaaatattgttgatgaagtggcttaccatgagaaatatccaac

aatctaccacttgagaaagaaactcgttgattctaccgataaagcagatctcaggttaatctaccttgctttggcacatatgataaagtttagaggacacttcttgattgagggagatcttaatcctgataa

ctcagatgttgataagctttttattcaacttgtgcagacatataaccaacttttcgaagagaatccaatcaacgcttcaggagttgatgctaaagcaatattgagtgcaagactctctaagtctaggagact

cgaaaatcttatagctcagttgcctggagaaaagaagaacggattattcggtaaccttatcgcactctctctcggtcttactccaaacttcaagtcaaacttcgatcttgctgaagatgcaaaattgcaact

ctctaaggatacatacgatgatgatttggataatctcttagctcagattggagatcaatatgcagatctttttcttgctgcaaaaaacctctcagatgctatccttttgagtgatatacttagagttaacactg

aaattacaaaggctcctctttcagcaagtatgatcaagagatacgatgagcatcaccaggatttgactctcttaaaggctttagttagacaacagcttccagaaaagtataaggagattttctttgatcagt

ctaagaacggatatgctggttacatcgatggaggtgcatctcaagaagagttctacaagttcattaaacctatcttggaaaagatggatggaaccgaagagcttttggttaaactcaacagagaggatct

cttaagaaagcagaggactttcgataacggatctatccctcatcaaatacaccttggtgaattgcatgctattttgaggagacaagaggatttctacccattcctcaaggataacagagaaaagattgag

aagattcttacctttaggatcccttattacgttggaccattggctaggggtaacagtaggttcgcatggatgactagaaaatctgaagagacaattaccccttggaattttgaagaggttgtggataaggg

tgcttctgcacagtcttttattgaaagaatgacaaacttcgataagaatttgcctaacgagaaagttctcccaaagcattctcttttgtacgaatacttcactgtgtacaatgagcttacaaaggttaaatat

gtgaccgaaggaatgagaaagccagcttttctttcaggagagcagaagaaagcaatcgttgatctcttattcaaaactaacagaaaggttacagtgaagcaacttaaggaagattacttcaagaaaat

cgagtgcttcgattctgttgaaatatcaggagtggaggatagattcaatgcttctttgggtacttatcatgatcttttgaagattatcaaggataaggattttcttgataatgaggagaacgaagatattcttg

aggatatcgttttgactctcacattattcgaagatagagagatgattgaagagaggttaaaaacatacgctcaccttttcgatgataaggttatgaaacaacttaagaggagaaggtataccggatgggg

tagattgtctaggaagctcatcaacggaataagagataaacagtcaggaaagactatcctcgatttcttaaagagtgatggattcgctaacagaaacttcatgcaattgatccatgatgattctctcacttt

taaagaagatattcaaaaggctcaggtttcaggacagggagatagtttgcatgagcacattgctaatctcgcaggatcacctgctattaagaagggtatattgcaaactgttaaggttgtggatgaactc

gttaaagtgatgggaagacataagccagagaatatagtgattgaaatggctagggagaaccaaactacacagaaaggtcaaaagaattctagagaaaggatgaaaagaatcgaagagggaattaa

ggagttgggttcacaaattctcaaagaacatcctgttgagaatacacaacttcagaacgaaaagctttacttgtattacttgcagaacggaagagatatgtatgtggatcaagagctcgatattaatagg

cttagtgattacgatgttgatcacatcgtgcctcagtcttttctcaaggatgattcaatagataacaaggttcttactagatcagataagaataggggaaagtctgataacgtgccatcagaagaggttgtt

aagaagatgaagaactactggagacagctcctcaacgctaagctcataactcaaagaaagttcgataatctcacaaaagctgaaaggggaggtctttctgagttggataaagcaggattcattaagag

acagttggttgaaactaggcaaattacaaaacatgtggctcaaatccttgattcaaggatgaatactaagtacgatgaaaacgataagttgataagagaggttaaggttattacactcaagagtaaact

tgtttctgatttcagaaaggatttccagttctacaaggtgagggagattaataactaccatcacgctcacgatgcatatcttaacgctgttgtgggaacagcattaatcaagaaataccctaagcttgaatc

tgagttcgtttacggagattacaaggtttacgatgtgagaaaaatgattgctaagtcagaacaagagattggtaaagctactgcaaagtactttttctatagtaacattatgaactttttcaaaaccgaaat

cactttggctaacggagagatcagaaagaggccactcatagaaaccaacggagaaactggagagattgtttgggataaaggtagagatttcgctacagttaggaaggtgctttctatgcctcaggttaa

catcgtgaagaaaacagaagttcaaaccggaggttttagtaaggagtctatattgccaaagagaaactcagataagctcattgctaggaagaaagattgggatcctaagaaatacggaggtttcgata

gtccaacagttgcttattctgtgttggttgtggcaaaggttgaaaagggaaaaagtaagaaacttaagtctgtgaaggagcttttgggtatcactattatggaaagatcttcattcgagaaaaaccctata

gatttccttgaagctaaaggatacaaggaggttaagaaagatttgataattaaactcccaaagtattcactcttcgaattagagaatggtagaaaaaggatgttagctagtgcaggagaacttcaaaag

ggtaacgagctcgctctcccttctaagtatgttaattttctttacttggcatctcattatgagaagcttaaaggttcaccagaagataatgagcaaaaacagcttttcgtggaacagcataagcactacttg

gatgaaatcatagagcaaatttcagaattttctaagagagttattttggctgatgcaaacttagataaggtgctttctgcttacaataagcatagagataagcctataagggaacaagcagagaacattat

ccaccttttttaccttgactaatctcggagctccagctgcttttaagtacttcgataccactatcgatagaaaaaggtatacatctaccaaggaagttttggatgcaactctcattcatcagtctatcaccggtc

tttatgagactagaattgatctttcacaacttggaggagataaaagaccagcagcaacaaagaaagcaggacaggctaaaaagaagaagtga

B.

>U6 promoter-gRNA-scaffold-U6 terminator

aagctttcgttgaacaacggaaactcgacttgccttccgcacaatacatcatttcttcttagcttttttttcttcttcttcgttcatacagtttttttttgtttatcagcttacattttcttgaaccgtagctttcgttttc

ttctttttaactttccattcggagtttttgtatcttgtttcatagtttgtcccaggattagaatgattaggcatcgaaccttcaagaatttgattgaataaaacatcttcattcttaagatatgaagataatcttca

aaaggcccctgggaatctgaaagaagagaagcaggcccatttatatgggaaagaacaatagtatttcttatataggcccatttaagttgaaaacaatcttcaaaagtcccacatcgcttagataagaaa

acgaagctgagtttatatacagctagagtcgaagtagtgattNNNNNNNNNNNNNNNNNNNNgttttagagctagaaatagcaagttaaaataaggctagtccgttatcaacttgaaaaa

gtggcaccgagtcggtgctttttttttgcaaaattttccagatcgatttcttcttcctctgttcttcggcgttcaatttctggggttttctcttcgttttctgtaactgaaacctaaaatttgacctaaaaaaaatct

caaataatatgattcagtggttttgtacttttcagttagttgagttttgcagttccgatgagataaaccaataactttgcttagatctaattcattccgttacacctctgatggagatggaaggttcttaataat

gatgccatttttgggtaataattttgaattagaatcaagggtataagattcataattaacatcacttaagcaaagttcgtaatatacgaccacaggatataatttttgaattca

Supplementary Fig. S1. Sequence of Arabidopsis codon-optimized *Cas9* gene with

two *NLSs* and U6 promoter-gRNA-scaffold-U6 terminator construct.

A. Arabidopsis codon-optimized *Cas9* gene was fused with one *NLS* at N terminus (red) and the other at C terminus (blue). *NLSs* were also Arabidopsis codon-optimized.

B. Sequence of U6 promoter-gRNA-scaffold-U6 terminator used in this study. U6 promoter was shown in orange, gRNA was shown in red, scaffold was shown in purple and U6 terminator was shown in green. Underlined part represents the sequence used for pCVA-gRNA in VIGE.

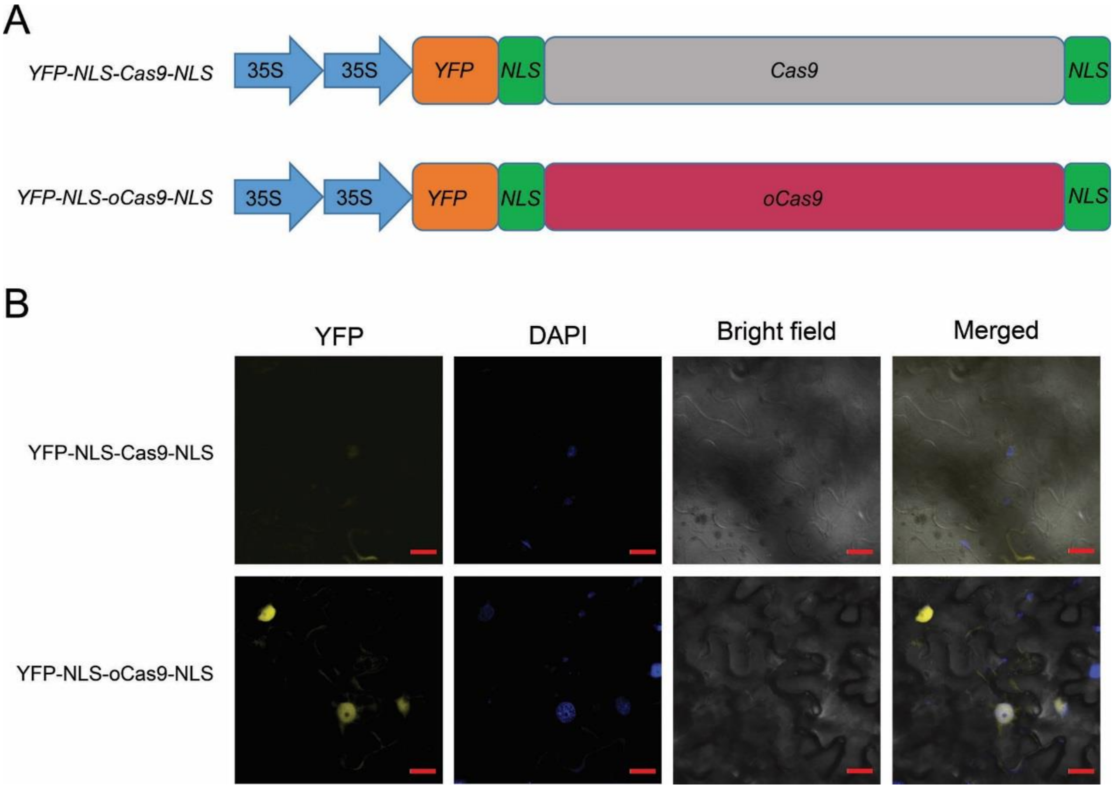

Supplementary Fig. S2. NLS-tagged oCas9 is localized in nucleus and showed higher expression level than non-codon-optimized Cas9.

A. Schematic diagram of Constructs YFP-NLS-oCas9-NLS and YFP-NLS-oCas9-NLS. **B**. Confocal images for subcellular localization of YFP-NLS-Cas9-NLS and YFP-NLS-oCas9-NLS. Bar = 20 μm.

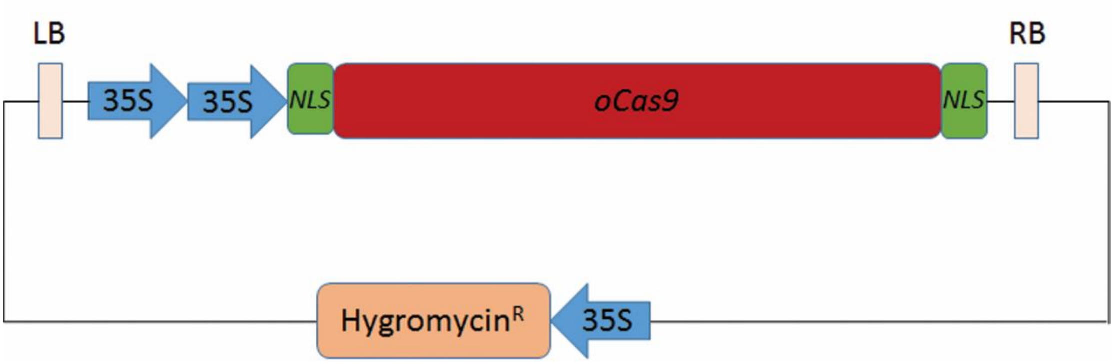

Supplementary Fig. S3. Schematic diagram of T-DNA construct used for generation of transgenic plants expressing oCas9. oCas9 contains two NLS at each terminus, and is expressed under control of CaMV 35S promoter with duplicated. Hygromycin resistance gene was used to select transgenic plants.

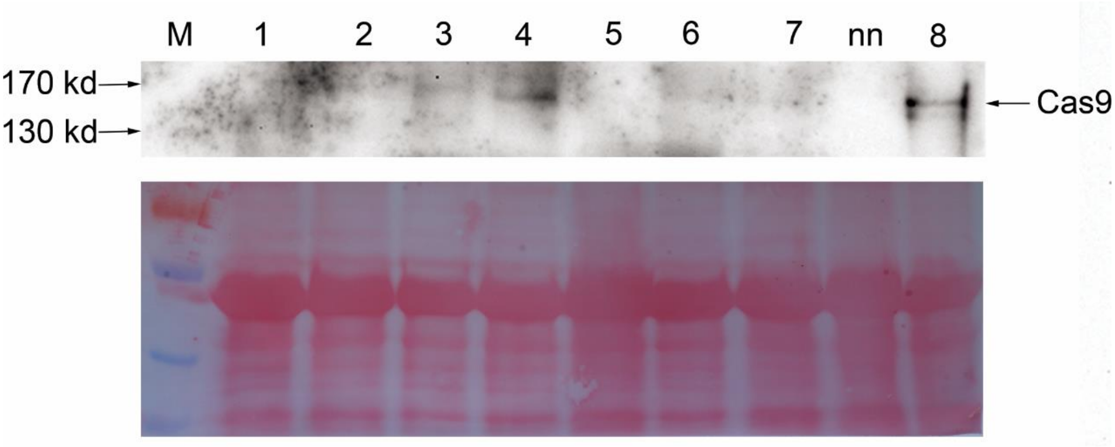

Supplementary Fig. S4. Western blot assay showed expression of Cas9 protein in KQ334 plants. Cas9 protein was detected by western blot using a monoclonal antibody against Cas9. Total protein from wildtype plants and 8 transgenic lines was extracted for western blot assays. The loading control was determined by Ponceau S staining showed at lower panel. M, protein marker. nn, wildtype *N. benthamiana* plant.

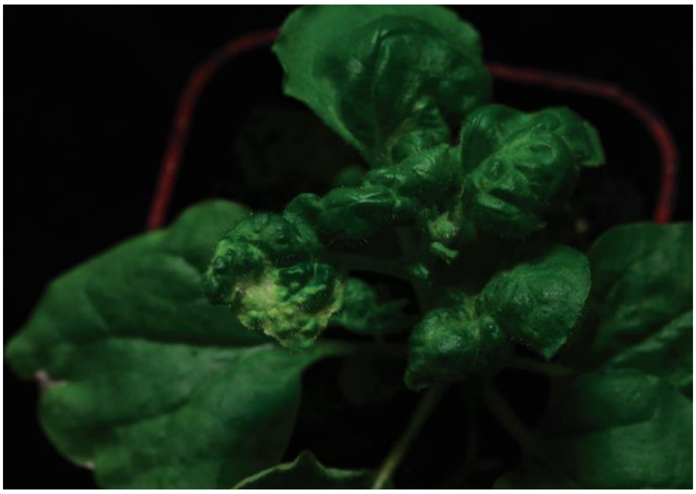

Supplementary Fig. S5. Phenotype of pCVA-gRNA::*NbPDS*/pCVB infected KQ334 plant at 3 wpi. KQ334 plant was agroinfected with CaLCuV carring gRNA::*NbPDS*. At 3 wpi, the infected plant showed photobleached phenotype on some part of the leaf as well as viral symptom, such as curly leaves.

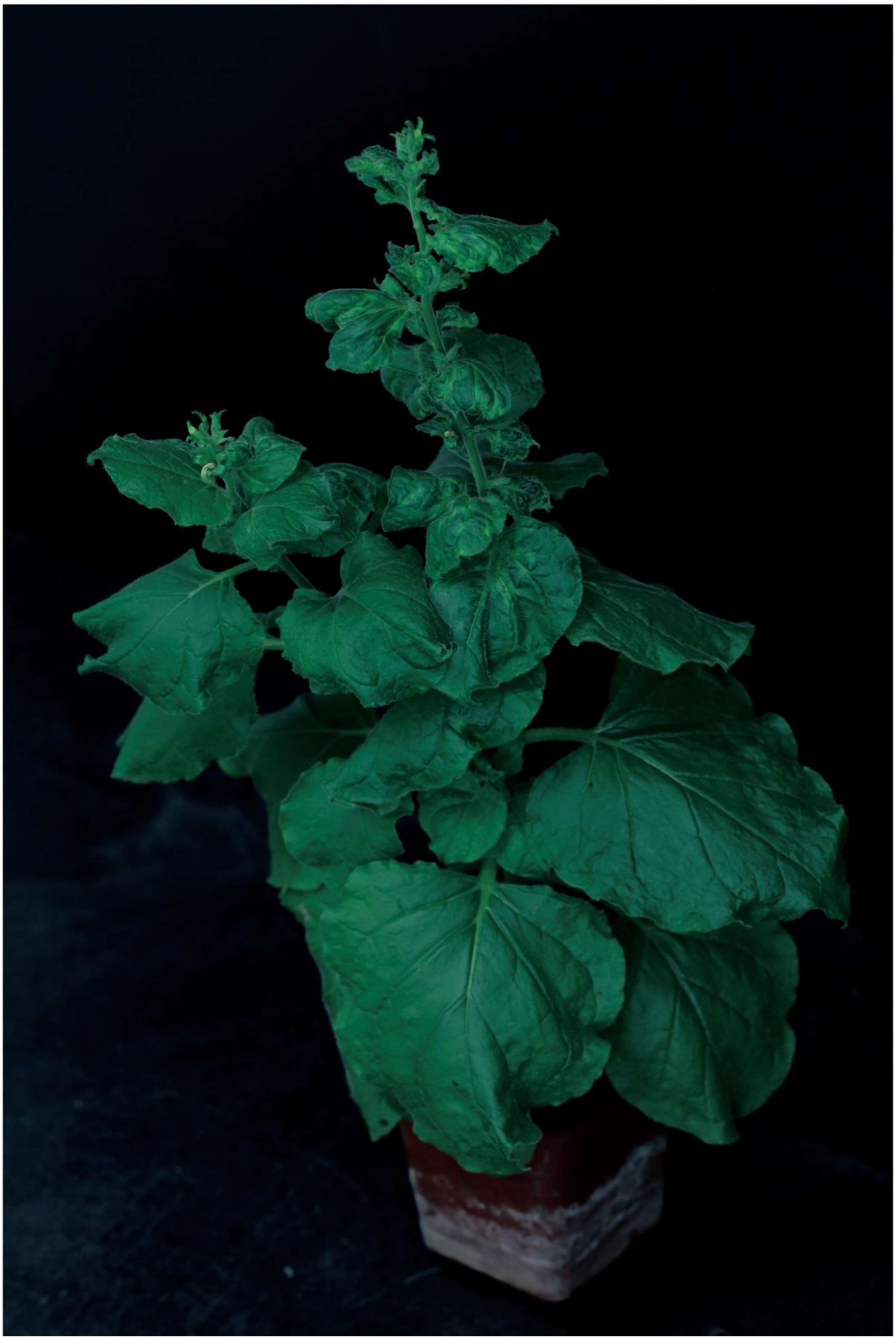

Supplementary Fig. S6.Agroinfection of wildtype *N. benthamiana* plants (without cas9 transgene) with pCVA-gRNA::*NbPDS*/pCVB did not cause a photo-bleached phenotype.

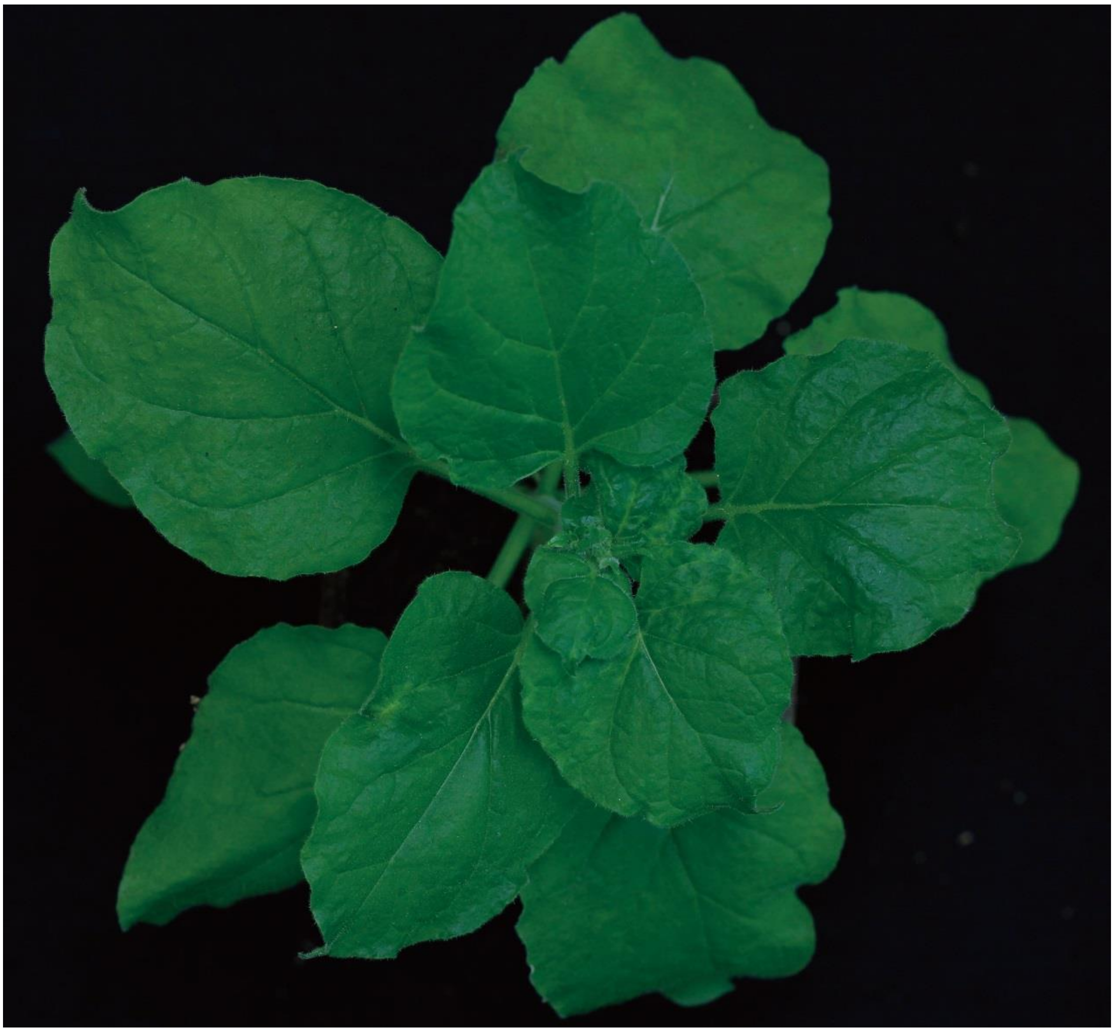

Supplementary Fig. S7. Phenotype of agroinfection of wildtype *N. benthamiana* plants (without cas9 transgene) with pCVA-gRNA::*NbIspH*/pCVB at 3 wpi. No photobleaching was observed.

Supplementary Table S1. List of primers used in this study.

List of primers used in plasmids construction, mutation assay and RT-PCR.

Name	Sequence (5'-3')	Brief description
oYK465	CgACgACAAgACCgTgACCATGGATTACAAGGATCACGATGGAGAT	For Cas9 to be cloned into binary vectors
oYK467	CgACgACAAgACCgTgATGGATTACAAGGATCACGATGGAGAT	
oYK468	gAggAgAAgAgCCgTtcaCTTCTTCTTTTTAGCCTGTCCTGCTTT	
oYK550	cccAAGCTTTCGTTGAACAACGGAAACT	For constructing U6 promoter-gRNA scaffold-U6 terminator
oYK551	gctatttctagctctaaaacAATCACTACTTCGACTCTAGCTGTA	
oYK552	gcaccgagtcggtgctttttTTTTGCAAAATTTTCCAGATCGATT	
oYK553	cccTGAATTCAAAAATTATATCCTGTGG	
oYK554	ctagagtcgaagtagtgattgttttagagctagaaatagc	
oYK555	atctggaaaattttgcaaaaaaaaagcaccgactcggtgc	
oYK615	GCGCTTCAAGGTGCACATGGgttttagagctagaaatagcaag	For U6::gRNA(targeting fsGUS) construction
oYK616	CCATGTGCACCTTGAAGCGCaatcactacttcgactctagctg	
oYK618	CGACGACAAGACCCTaccatgGCGCTTCAAGGTGCACATGGAGGTTACGTCCTGTAGAAACCCcaa	For 35S::*fsGUS* cloning
oYK619	GAGGAGAAGAGCCCTTCATTGTTTGCCTCCCTGCTGC	
oYK698	CGACGACAAGACCCTaccatgG	For identification of *fsGUS* mutation by Cas9
oYK701	AACTGCCTGGCACAGCAATTGC	
oYK1013	cccTCTAGAgttttagagctagaaatagc	For cloning pCVA-scaffold, pCVA-gRNA::*NbPDS*, pCVA-gRNA::*NbIspH*
oYK1056	gccgttaatttgagagtccagttttagagctagaaatagcaag	
oYK1057	tggactctcaaattaacggcaatcactacttcgactctagctg	
oYK1191	cccTCTAGAaagctttcgttgaacaacgg	
oYK1192	cccGGTACCaaaaaaagcaccgactcggtgccac	
oYK1380	GAATGGATATGAGTACACTTgttttagagctagaaatagcaag	
oYK1381	AAGTGTACTCATATCCATTCaatcactacttcgactctagctg	
oYK 1202	gccgttaatttgagagtcca	For testing gRNA expression by RT-PCR
oYK 1203	gcaccgactcggtgccacttt	
oYK 1483	GAATGGATATGAGTACACTT	
oYK 1469	GGAAATCGTGGAGGTGTG	For amplifying *IspH* locus including the gRNA target
oYK 1470	GTAGGGAAGAAGGAGCAA	
